# Impact of acute hyperglycemia on layer-specific left ventricular strain in asymptomatic diabetic patients: an analysis based on two-dimensional speckle tracking echocardiography

**DOI:** 10.1186/s12933-019-0876-3

**Published:** 2019-06-03

**Authors:** Jelena Bogdanović, Milika Ašanin, Gordana Krljanac, Nebojša M. Lalić, Aleksandra Jotić, Sanja Stanković, Nataša Rajković, Ljubica Stošić, Iva Rasulić, Jelena Milin, Dragana Popović, Ljiljana Bogdanović, Katarina Lalić

**Affiliations:** 1 Clinic for Endocrinology, Diabetes and Metabolic Diseases, Clinical Center of Serbia, dr Subotića 13, Belgrade, 11000 Serbia; 2 Faculty of Medicine, University of Belgrade, dr Subotića 8, Belgrade, 11000 Serbia; 3 Clinic for Cardiology, Clinical Center of Serbia, Pasterova 2, Belgrade, 11000 Serbia; 4 Center for Medical Biochemistry, Clinical Center of Serbia, Pasterova 2, Belgrade, 11000 Serbia; 5 Institute for Medical Statistics and Informatics, dr Subotića 15, Belgrade, 11000 Serbia; 6 Institute for Pathology, dr Subotića 1, Belgrade, 11000 Serbia

**Keywords:** Diabetes, Acute hyperglycemia, Left ventricle, Multilayer strain, Two-dimensional speckle tracking

## Abstract

**Background:**

Hyperglycemia has detrimental effect on ischemic myocardium, but the impact of acute hyperglycemia on the myocardium in asymptomatic diabetic patients has not been fully elucidated. Thus, this follow-up study was aimed to investigate the effects and reversibility of acute hyperglycemia on regional contractile function of left ventricle (LV) in diabetic patients without cardiovascular disease.

**Methods:**

The two-dimensional speckle tracking echocardiography (2D-STE), including multilayer strain analysis, was used for evaluation of global and regional LV function in asymptomatic, normotensive patients with uncomplicated diabetes, with acute hyperglycemia ( ≥ 11.1 mmol/l) (Group A, n = 67), or with optimal metabolic control (fasting plasma glucose < 7 mmol/l and HbA1c < 7%) (Group B, n = 20), while 20 healthy individuals served as controls (Group C). In group A, after 72 h of i.v. continuous insulin treatment (at the time euglycemia was achieved) (second examination) and after 3 months following acute hyperglycemia (third examination) 2D-STE was repeated.

**Results:**

Global longitudinal strain (GLS) (− 19.6 ± 0.4%) in Group A was significantly lower in comparison to both groups B (− 21.3 ± 0.4%; p < 0.05) and C (− 21.9 ± 0.4%; p < 0.01) at baseline, while we could not detect the differences between groups B and C. Peak systolic *longitudinal *endocardial (Endo), mid-myocardial (Mid) and epicardial (Epi) layer strain were significantly lower in group A at baseline compared to both groups B and C. Deterioration in peak systolic *circumferential* strain was observed at basal LV level, in all three layers (Endo, Mid and Epi) and in mid-cavity LV level in Epi layer in group A in comparison to group C. Moreover, in group A, after euglycemia was achieved (at second and third examination) GLS, as well as peak longitudinal and circumferential strain remain the same.

**Conclusion:**

Acute hyperglycemia in asymptomatic diabetic patients has significant negative effects on systolic LV myocardial mechanics primarily by reducing GLS and multilayer peak systolic longitudinal and circumferential strain which was not reversible after three months of good glycemic control.

## Background

Diabetes, as a chronic metabolic disease, is characterized by elevated blood glucose levels (hyperglycemia) that can lead to specific vascular complications, including heart failure, while cardiovascular complications are the leading cause of morbidity and mortality in these patients [1, 2]. A sudden increase of blood glucose levels (acute hyperglycemia) has been documented to occur in people with previously diagnosed diabetes or impaired glucose tolerance. Acute hyperglycemia is usually defined according to Ishihara [3] as blood glucose levels which are at hospital admission measured to be ≥ 11.1 mmol/l. Over the years, numerous studies have shown that acute hyperglycemia has many harmful effects on the cardiovascular system. It causes insufficiency, dysregulation and overall endothelial dysfunction [4] and reduces: collateral circulation [5], spontaneous reperfusion in patients with acute myocardial infarction (AMI) with ST-segment elevation [6] and it deepens the QT [7]. In addition, some studies have shown that, during AMI, acute hyperglycemia is associated with impaired function (dysfunction) of the left ventricle (LV) [8, 9].

Recently, a new two-dimensional speckle tracking echocardiography (2D-STE) has been developed which enables reliable assessment of regional deformation in three directions: longitudinal, circumferential and radial. With the use of this technique it is possible to measure myocardial function parameters and quantify global and regional systolic and diastolic myocardial function [10]. Also, this technique allows to track and display myocardial deformation through its’ different layers [11]. In that context, it has been previously shown that the magnitude of LV longitudinal strain at the basal level decreases, whereas the apical LV longitudinal strain increases with increasing age, while presence of diabetes modulate the effect of age on LV strain [12]. Similarly, hypertension and obesity, both in asymptomatic diabetic and nondiabetic patients induce sequential impairment of LV function from the endocardium to the epicardium [13, 14]. Finally, in asymptomatic, normotensive diabetic patients (both type 1 and type 2) with normal LVEF significant subclinical LV systolic dysfunction was found [15, 16]. Therefore, layer-specific evaluation of LV strain could provide a better understanding of the effect of acute hyperglycemia on LV systolic function in asymptomatic diabetic patients.

Most previous research is focused on assessing impacts that acute hyperglycemia has on the myocardium during AMI and there is no study concerning it’s effects on the contractile function and any early abnormalities of the heart, determined using 2D-STE, in diabetic patients without previous cardiovascular disease (CVD). Thus, the aim of this study was to investigate the effects of acute hyperglycemia on regional function of the LV, as well as to determine the reversibility of possible myocardial changes following euglycemia in diabetic patients who do not present any symptoms and clinical signs of CVD.

## Material and methods

### Study population

A total of 67 patients with diabetes (*Group A*), 30 with type 1 diabetes (T1D) and 37 with type 2 diabetes (T2D), both gender (45 males), with an average age of 38.3 ± 1.6 years that had acute hyperglycemia (plasma glucose ≥ 11.1 mmol/l) were included in this prospective study which was performed over a period of 4 years (from January 2014 to January 2018). Patients included in this study were carefully selected from the patients admitted to Intensive Care Unit (ICU) according to the following inclusion criteria: (1) previously known diabetes (32 patients, 47.8%) or newly onset diabetes (35 patients, 52.2%) with hyperglycemia (plasma glucose ≥ 11.1 mmol/l) on the admission; (2) free of previously known or newly diagnosed CVD (including hypertension) or on treatment for CVD or hypertension; (3) free of known or newly diagnosed microvascular diabetic complications. Those patients younger than 18 or older than 65 years of age, with unstable angina pectoris, AMI or history of coronary interventions, heart failure, atrial fibrillation or irregular rhythm on ECG, malignant diseases, obstructive pulmonary disease, hepatic or renal failure (eGFR ≤ 60 ml/min/1.73 m^2^), acute or chronic infections, ketoacidosis, treated with corticosteroids or immunosuppressive agents and with new uncontrolled hypertension (blood pressure higher than 180/110 mmHg) requiring treatment were excluded from the study.

For comparison, there were two control groups: (1) *Group B *(n = 20, 10 males, 43.2 ± 3.6 years old) consisting of carefully selected patients with diabetes who had optimal metabolic control (HbA1c ≤ 7.0%) with fasting plasma glucose < 7.0 mmol/l at the time of inclusion in the study and without any known or newly diagnosed micro- or macrovascular diabetic complications; (2) *Group C* consisting of healthy volunteers (n = 20, 8 males, 36.2 ± 2.0 years old).

This study has been approved by the Institutional Ethics Committee and all patients have signed the informed consent form prior to inclusion. All of the investigated patients (Group A) were admitted to the ICU of the Clinic for Endocrinology, Diabetes and Metabolic Diseases, Clinical Center of Serbia, and main reason for admission was hyperglycemia. The echocardiographic examination was exerted by experienced cardiologist in the Clinic for Cardiology, Clinical Center of Serbia, while all biological samples were processed at Center for Medical Biochemistry, Clinical Center of Serbia.

### Research design

Upon the admission to ICU in all investigated patients (Group A) a full medical history was obtained and physical exam with anthropometric measurements were undertook. Body mass index (BMI) was calculated according to the standard formula (kg/m^2^) from body weight and height measured with a digital scale. Intravenous cannulas were inserted in antecubital vein of both arms for blood sampling, as well as for saline, glucose and insulin infusion for the next 72 h. First, blood was drowning for basal laboratory analysis and immediately after that all patients underwent a resting standard echocardiographic examination before any antihyperglycemic therapy was started.

All investigated patients were subjected to the same therapeutic protocol until the point when euglycemia (plasma glucose ≤ 6.0 mmol/l) was achieved (by the 72 h). Patients were treated with a solution of rapid acting human insulin (Actrapid, Novo Nordisk, Denmark) dissolved in a physiological saline solution (Hemofarm, Vršac, Serbia) (1 IU of insulin in 1 ml of NaCl 0.9% saline solution) and apply by using continuous insulin infusion pump (Perfusor Space Infusion, BBraun, Germany) starting at a rate of 6 IU/h. Glycemic values were taken every hour (AccuChek glucometer, Roche, Germany) and infusion rate was further adjusted according to the glucose levels using the standard protocol in order to achieve near-normal glycemia. Additional saline salts, 5% glucose solution, electrolyte replacement and antibiotics were also implemented i.v. At the time euglycemia was achieved (after 72 h of i.v. continuous insulin treatment) and after three months ( ± 15 days) following acute hyperglycemia biochemical analyses and echocardiography examinations (done by the same cardiologist) were repeated.

Subjects in control groups (Groups B and C) were examined only once when a medical history data was obtained together with anthropometric parameters, laboratory tests and an echocardiogram was performed.

### Echocardiographic examination

All resting standard echocardiographic examinations were performed using Vivid E9 (General Electric). Data were acquired with a 3.5 MHz transducer in the parasternal (long- and short-axis views) and apical views (two- and four-chamber and apical long-axis views). Echocardiographic methods as M-mode, 2D, color Doppler, pulse Doppler and continue Doppler were performed. All definitions and rule for measurements were in accordance with the recommendations of the European and American Society of Echocardiography [17]. The LV end-diastolic (EDV) and end-systolic volumes (ESV) were measured from the apical two- and four-chamber views, and LV ejection fraction (EF) was calculated using the Simpson’s rule [17]. LV diastolic function was evaluated using early (E-wave) and late (A-wave) transmitral velocities, the E/A ratio and the E-deceleration time (DT) obtained by the spectral pulsed-wave Doppler recordings and by tissue Doppler imaging. The peak early diastolic velocity (E′) was measured at the basal myocardial segments, on the apical four-chamber view, at mitral annulus level (Em′ for the septal and El′ for the lateral) and average value was considered. Finally E/E′ ratio was calculated [17].

### 2D left ventricular speckle-tracking strain analyses

**Fig. 1:**
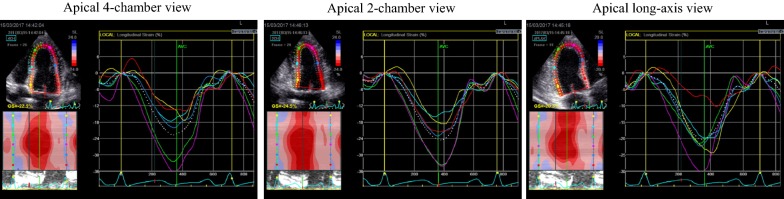
An example of a color-coded two-dimensional speckle tracking echocardiography (2D-STE) of the left ventricle (LV) and corresponding time–strain curves from 18 LV segments derived from the apical 4-, 2-chamber and long-axis views for measurement of global longitudinal strain (GLS) in diabetic patient with acute hyperglycemia. GLS was calculated as the average peak strain of 18 LV segments, and in this example the value of GLS is − 22.4%

**Fig. 2:**
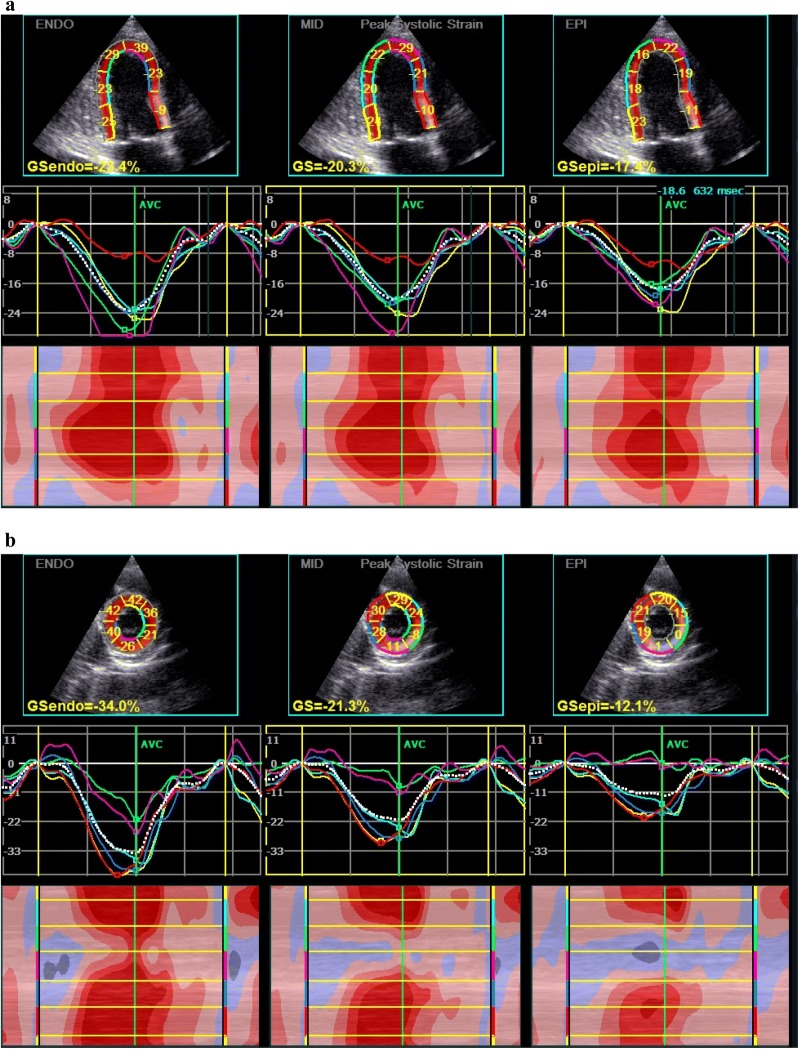
Multilayer longitudinal and circumferential strains determined by two-dimensional speckle tracking echocardiography (2D-STE) in diabetic patient with acute hyperglycemia. **a **Subendocardial (Endo), mid-myocardial(Mid) and subepicardial(Epi) layers of longitudinal strain assessed in apical long-axis views; **b **Endo, Mid and Epi layers of circumferential strain at base (mitral valve) evaluated in parasternal short-axis view

The two-dimensional speckle tracking echocardiography (2D-STE), as noninvasive ultrasound imaging technique was used for an objective and quantitative evaluation of global and regional myocardial function. The recordings with a frame rate between 50 and 70 frames/s were performed and analysed offline using General Electric software (EchoPAC software version 113 GE Medical Systems). Standard 2D gray-scale images of the LV were acquired at apical four-, two-chambers and apical long-axis view (Fig. 1), as well as parasternal short-axis view at base (mitral valve), papillary muscles and axis level. Global longitudinal LV myocardial strain (GLS) and systolic longitudinal, circumferential and radial strain were calculated offline. Longitudinal, circumferential and radial strain is analyzed on the 18-segment segmentation model. However, radial strain was not further processed because of a problem using 2D acquisition methods or a low number of high-quality images and suboptimal tracking which influenced the consistency of findings. Multilayer longitudinal and circumferential strains were determined by 2D-STE software which automatically creates a region of interest, which contained subendocardial (Endo), mid-myocardial (Mid) and subepicardial (Epi) layers (Fig. 2). Multilayer longitudinal strain was assessed in apical four-chamber, two-chamber and long-axis views, whereas multilayer circumferential strain was evaluated in the short-axis at the three levels as mentioned [18]. In randomly selected 15 patients, 2–4 weeks after performing the initial measurements, strain analysis was repeated by the same observer (G.K.) to determine intra-observer reproducibility. The absolute difference between two measurements divided by the average value of these two measurements was calculated together with interclass correlation coefficient (ICC).

### Laboratory analyses

Plasma glucose concentration (commercial test reagent, Abbott Diagnostic) and HbA1c levels (commercial test reagent, SEBIA, France) were determined by spectrophotometry. Serum lipid levels (total and HDL cholesterol (Ch) and triglycerides) were analyzed enzymatically using commercial kit (ADVIA Chemistry), while LDL-Ch was calculated by standard Friedewald formula. Serum concentrations of troponin (commercial test reagent Roche Diagnostics) and N terminal brain fragment (B-type) for natriuretic peptide (NT-proBNP; commercial test reagent Abbott Diagnostics) were determined by the CLIA test.

### Statistical analysis

All continuous variables are presented as mean ± SE, while categorical data are presented as percentages. Sample size for the study was calculated according to previously published results using standard methods with probability (power) 0.8 and type I error probability 0.05 and we estimated that minimum number of investigated patients (Group A) should be 61. The differences between the groups were tested using a one-way analysis of variance (ANOVA) with Bonferroni post hoc test, while Kruskal–Wallis test was used for categorical variables. The statistical analyses were performed using SPSS software, version 20.0 (SPSS Inc., USA). The level of statistical significance was set at *p* < *0.05*.

## Results

### Characteristics of the study populations

Baseline demographic and anthropometric characteristics of the investigated subjects are presented in Table 1. All three groups did not differ in respect to gender, age, body weight and BMI. Proportion of patient with T1D and T2D was similar in group A and group B, as well as average duration of diabetes. The analysis of the previous medication revealed similar pattern the drugs used related to diabetes (oral hypoglycemic agents or insulin), while none of the investigated subjects used any cardiovascular drugs.

**Table 1:** Baseline demographic and anthropometric characteristics of investigated patients

Variables	Group A (DM with acute hyperglycemia) n = 67	Group B (DM with euglycemia) n = 20	Group C (healthy controls) n = 20	p value
Gender (male), n (%)	45 (67.2)	10 (50.0)	8 (40.0)	0.065
Age (years)	38.3 ± 1.6*	43.2 ± 3.6	36.2 ± 2.0	0.208
Body weight (kg)	79.6 ± 2.6	73.6 ± 2.8	72.0 ± 3.6	0.345
BMI (kg/m^2^)	25.7 ± 0.8	24.6 ± 1.0	23.8 ± 0.7	0.209
Diabetes				
Type 1, n (%)	30 (44.8)	8 (40.0)	–	0.705
Type 2, n (%)	37 (55.2)	12 (60.0)	–	
Diabetes duration (years)	8.8 ± 1.2	8.4 ± 1.2	–	0.798
Therapy, n (%)				
No previous therapy	33 (49.3)	0	–	
OAD	11 (16.4)	8 (40.0)	–	0.570
Insulin	23 (34.3)	12 (60.0)	–	

*DM* diabetes mellitus, *BMI* body mass index, *OAD* oral anti diabetes drugs

* Data are expressed as mean ± SE

### Laboratory measurements

The biochemistry analyses performed in the investigated patient groups are shown in Table 2. At baseline, we found significantly higher blood glucose levels in group A (upon hospital admission; first examination) than in both groups B and C (p < 0.001, respectively), while there were no differences when we compared groups B and C. After 72 h hours of insulin treatment in group A (second examination) we achieved normalization of the glycemic values which slightly increase after three months (third examination) (p < 0.001). Similarly, HbA1c level were significantly higher in group A in comparison to both groups B and C (p < 0.001, respectively) and significantly decreased after three months in group A (p < 0.001).

**Table 2:** Biochemistry analyses in diabetic patients with acute hyperglycemia (*Group A*) at baseline (upon hospital admission), second (after euglycemia was achieved, 72 h) and third examination (after 3 months), in diabetic patients with euglycemia (*Group B*) and in healthy controls (*Group C*)

	Group A			p value*	Group B	Group C	p value**
	Upon hospital admission	Second examination	Third examination				
Blood glucose (mmol/l)	22.5 ± 1.1^a^	5.5 ± 0.1	7.2 ± 0.2	< 0.001	5.8 ± 0.2	5.1 ± 0.1	< 0.001
HbA1c (%)	11.5 ± 0.3	NA	7.9 ± 0.2	< 0.001	6.1 ± 0.1	5.0 ± 0.1	< 0.001
Total Ch (mmol/l)	6.2 ± 0.4	5.4 ± 0.2	5.1 ± 0.2	0.002	4.8 ± 0.2	5.1 ± 0.2	0.055
HDL-Ch (mmol/l)	1.2 ± 0.1	1.1 ± 0.1	1.3 ± 0.1	0.053	1.4 ± 0.1	1.6 ± 0.1	< 0.001
LDL-Ch (mmol/l)	2.9 ± 0.2	3.0 ± 0.1	2.8 ± 0.1	0.039	2.7 ± 0.1	2.8 ± 0.2	0.747
Triglyceride (mmol/l)	6.4 ± 1.4	3.2 ± 0.4	2.3 ± 0.2	0.003	1.9 ± 0.2	1.5 ± 0.3	0.036
Troponin (ng/l)	8.9 ± 0.8	6.8 ± 0.5	6.1 ± 0.6	0.013	8.2 ± 0.7	7.6 ± 1.1	0.652
NT-proBNP (pg/ml)	25.0 (28.5)^b^	14.0 (17.0)	15.0 (20.0)	< 0.001	21.5 (41.5)	19.0 (25.8)	0.597

*Ch* cholesterol, *NT-proBNP* N-terminal pro b-type natriuretic peptide

* p—Repeated measures ANOVA (upon hospital admission, second examination and third examination)

** p—One way ANOVA between Group A (upon hospital admission), Group B and Group C. Post Hoc Bonferroni test between the groups is described in the text

^a^Data are presented as mean ± SE or as ^b^median

Regarding the lipid parameters, total and LDL-Ch at baseline were similar between the groups, although we found in group A significantly lower levels of both total and LDL-Ch levels at second and third examinations (p < 0.05). In contrast, HDL-Ch was significantly lower at baseline in group A vs both groups B and C (p < 0.001, respectively) and remain unchanged at second and third examinations in group A, while we could not find any differences between groups B and C.

Also, triglycerides value was significantly higher at baseline when we compared group A with both groups B and C (p < 0.05, respectively). In group A, triglycerides significantly decreased at second examination (p < 0.05) and remain unchanged at third examination (after three months).

Simultaneously, the levels of troponin and NT-proBNP were similar across all three groups at baseline, while we registered significant lowering of both parameters at second and third examination in group A (p < 0.05 and p < 0.001, respectively).

### Conventional echocardiography

LV EDV and ESV, LV EF, as well as left atrium volume index (LAVI) were similar between the groups at baseline. Also, there were no changes in all those parameters in group A at second and third examinations (Table 3).

**Table 3:** Echocardiographic parameters in diabetic patients with acute hyperglycemia (*Group A*) at baseline (upon hospital admission), second (after euglycaemia was achieved, 72 h) and third examination (after 3 months), in diabetic patients with euglycemia (*Group B*) and in healthy controls (*Group C*)

	Group A			p value*	Group B	Group C	p value**
	Upon hospital admission	First examination	Second examination				
IVS, cm	0.94 ± 0.01^a^	0.97 ± 0.01	0.98 ± 0.01	0.521	0.96 ± 0.03	0.95 ± 0.04	0.681
LVEDV, ml	81.9 ± 3.1	85.6 ± 3.0	85.8 ± 3.0	0.165	78.8 ± 5.0	80.9 ± 4.6	0.877
LVESV, ml	33.1 ± 1.6	33.7 ± 1.5	33.9 ± 1.6	0.828	31.5 ± 2.5	29.8 ± 2.0	0.561
LVEF, %	62.8 ± 0.6	63.4 ± 0.4	63.2 ± 0.4	0.507	63.6 ± 0.8	65.1 ± 0.4	0.075
LAVI, mL/m^2^	17.3 ± 0.5	17.6 ± 0.6	17.9 ± 0.4	0.604	17.2 ± 0.9	16.9 ± 1.0	0.343
E, m/s	0.7 ± 0	0.7 ± 0	0.7 ± 0	0.501	0.6 ± 0	0.8 ± 0	0.001
A, m/s	0.6 ± 0	0.6 ± 0	0.6 ± 0	0.115	0.6 ± 0	0.6 ± 0	0.290
E/A ratio	1.2 ± 0.1	1.2 ± 0.1	1.2 ± 0.1	0.815	1.0 ± 0.1	1.4 ± 0.1	0.002
E/E′	6.1 ± 0.2	6.0 ± 0.2	6.0 ± 0.2	0.861	5.4 ± 0.3	5.5 ± 0.4	0.139
DT, ms	223.9 ± 5.7	208.8 ± 4.8	206.9 ± 4.8	0.001	219.7 ± 6.9	214.9 ± 7.3	0.683

*IVS* interventricular septum, *LVEDV* left ventricular end-diastolic volume, *LVESV* left ventricular end-systolic volume, *LVEF* left ventricular ejection fraction, *LAVI* left atrial volume index, *E* early wave, *A* atrial wave, *E′* early diastolic mitral annulus velocity, *DT* deceleration time

* p—Repeated measures ANOVA: upon hospital admission, second examination (after euglycemia was achieved, 72 h) and third examination (after 3 months)

** p—One way ANOVA between Group A (upon hospital admission), Group B and Group C. Post Hoc Bonferroni test between the groups is described in the text

^a^Data are presented as mean ± SE

When we analyzed LV diastolic function we found that both diabetes groups A and B have significantly lower transmitral E wave and E/A ratio compared to healthy controls (group C), while we did not find any differences in transmitral A wave. E/E’ ratio had a trend to be higher in group A compared to both groups B and C at baseline, but did not reach statistical significance. Additionally, in group A, observed lower E and E/A ratio remained unchanged after euglycemia was achieved (second examination) or after 3 months (third examination) (Table 3). In contrast, although there were no significant differences in DT across the groups at baseline, we found that in group A, DT was significantly shorter at both second and third examinations in comparison to baseline (p < 0.001) (Table 3).

### 2D LV speckle-tracking strain analyses

**Fig. 3:**
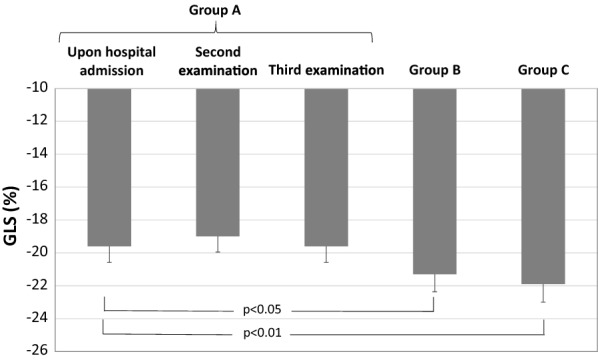
Global longitudinal strain (GLS) in diabetic patients with acute hyperglycemia (*Group A*) at baseline (upon hospital admission), second (after euglycemia was achieved, 72 h) and third examination (after 3 months), in diabetic patients with euglycemia (*Group B*) and in healthy controls (*Group C*)

We found significantly lower GLS (− 19.6 ± 0.4%) in Group A in comparison to both groups B (− 21.3 ± 0.4%; p < 0.05) and C (− 21.9 ± 0.4%; p < 0.01) at baseline, while we could not detect the differences between groups B and C. Moreover, in group A, after euglycemia was achieved (second examination) we did not find any changes in GLS (− 19.0 ± 0.3%; p = ns) which remain the same at third examination (after 3 months) (− 19.6 ± 0.3%; p = ns) (Fig. 3).

When we analyzed peak systolic *longitudinal* strain, we detected that Endo, Mid and Epi myocardial layers strain are significantly lower in group A at baseline compared to both groups B and C (Table 4). In addition, observed lower Endo, Mid and Epi peak systolic longitudinal strain in group A remain unchanged at second and third examinations (Table 5).

**Table 4:** 2D left ventricular speckle-tracking strain analyses in diabetic patients with acute hyperglycemia (*Group A*) at baseline (upon hospital admission), in diabetic patients with euglycemia (*Group B*) and in healthy controls (*Group C*)

	Group A	Group B	Group C	p value	Post Hoc Bonferroni test (p)		
					AvsC	AvsB	BvsC
Peak systolic longitudinal strain (%)							
Endo	− 22.8 ± 0.4^a^	− 24.7 ± 0.4	− 25.5 ± 0.5	< 0.001	0.001	0.023	1.000
Mid	− 19.7 ± 0.3	− 21.1 ± 0.4	− 22.1 ± 0.4	< 0.001	0.001	0.075	0.577
Epi	− 17.4 ± 0.3	− 18.8 ± 0.3	− 19.3 ± 0.4	0.001	0.002	0.042	1.000
Peak systolic circumferential strain (%)							
Basal LV level							
Endo	− 20.0 ± 0.8	− 23.2 ± 1.2	− 24.2 ± 1.1	0.010	0.023	0.127	1.000
Mid	− 13.8 ± 0.6	− 16.5 ± 0.9	− 17.4 ± 0.9	0.002	0.005	0.043	1.000
Epi	− 9.5 ± 0.4	− 11.3 ± 0.6	− 12.8 ± 0.8	< 0.001	< 0.001	0.091	0.442
Mid-cavity LV level							
Endo	− 27.6 ± 0.9	− 29.1 ± 1.0	− 29.2 ± 1.1	0.499	–	–	–
Mid	− 19.5 ± 0.7	− 20.7 ± 0.7	− 21.4 ± 1.0	0.262	–	–	–
Epi	− 13.5 ± 0.5	− 15.1 ± 0.6	− 16.0 ± 1.0	0.038	0.049	0.393	1.000
Apical LV level							
Endo	− 35.7 ± 1.0	− 34.1 ± 1.7	− 35.5 ± 1.6	0.713	–	–	–
Mid	− 26.9 ± 0.8	− 25.6 ± 1.4	− 26.8 ± 1.4	0.727	–	–	–
Epi	− 20.7 ± 0.7	− 18.9 ± 1.3	− 20.9 ± 1.3	0.461	–	–	–

*Endo* subendocardial layer, *Mid* mid-myocardial layer, *Epi*: subepicardial layer, *LV* left ventricular

p—One way ANOVA between Group A (upon hospital admission), Group B and Group C

^a^Data are presented as mean±SE

**Table 5:** 2D left ventricular speckle-tracking strain analyses in diabetic patients with acute hyperglycemia (*Group A*) at baseline (upon hospital admission), second (after euglycemia was achieved, 72 h) and third examination (after 3 months)

	Upon hospital admission	Second examination	Third examination	p value*
Peak systolic longitudinal strain (%)				
Endo	− 22.8 ± 0.4^a^	− 22.4 ± 0.3	− 23.1 ± 0.4	0.334
Mid	− 19.7 ± 0.3	− 19.2 ± 0.3	− 19.9 ± 0.3	0.201
Epi	− 17.4 ± 0.3	− 16.9 ± 0.3	− 17.6 ± 0.3	0.132
Peak systolic circumferential strain (%)				
Basal LV level				
Endo	− 20.0 ± 0.8	− 20.7 ± 0.8	− 20.2 ± 1.3	0.681
Mid	− 13.8 ± 0.6	− 14.7 ± 0.7	− 15.2 ± 0.8	0.120
Epi	− 9.5 ± 0.4	− 10.6 ± 0.5	− 10.5 ± 0.5	0.071
Mid-cavity LV Level				
Endo	− 27.6 ± 0.9	− 27.3 ± 0.8	− 27.0 ± 0.7	0.773
Mid	− 19.5 ± 0.7	− 19.3 ± 0.7	− 19.6 ± 0.7	0.906
Epi	− 13.5 ± 0.5	− 13.9 ± 0.6	− 14.5 ± 0.6	0.293
Apical LV level				
Endo	− 35.7 ± 1.0	− 33.4 ± 0.9	− 33.1 ± 0.8	0.103
Mid	− 26.9 ± 0.8	− 25.2 ± 0.8	− 24.9 ± 0.7	0.155
Epi	− 20.7 ± 0.7	− 19.5 ± 0.6	− 19.1 ± 0.7	0.172

*Endo* subendocardial layer, *Mid* mid-myocardial layer, *Epi* subepicardial layer, *LV* left ventricular

* p—Repeated measures ANOVA: upon hospital admission, second and third examination

^a^Data are presented as mean±SE

Similarly, we also found deterioration in peak systolic *circumferential* strain: at basal LV level, all three layers strain (Endo, Mid and Epi) were significantly lower in group A in comparison to group C, while only Mid layer strain was lower in comparison to both groups B and C (Table 4). However, identified changes in group A have not improved at second (after euglycemia achieved) or third examination (after 3 months) (Table 5). In contrast, at mid-cavity LV level, only in Epi layer we found significantly lower peak systolic circumferential strain in group A vs group C, but not vs group B, which remain unchanged during the follow up period (Tables 4, 5). In apical LV level, there were no difference in peak systolic circumferential strain at any layer (Endo, Mid and Epi) among the investigated groups (Table 4).

The intra-observer variability for longitudinal and circumferential strain was 4.2% and 5.1% (respectively). ICC for intra-observer variability of the corresponding values was 0.899 (95% CI 0.607–0.969; p < 0.001) and 0.721 (95% CI 0.220–0.904; p < 0.01).

## Discussion

Results from this study indicate that acute hyperglycemia (plasma glucose ≥ 11.1 mmol/l) in diabetic patients without any CVD and with preserved LVEF have significant negative effects on systolic LV function primarily by reducing GLS, peak systolic longitudinal (at Endo, Mid and Epi layers) and circumferential strain (at all three layers in basal level). Moreover, observed changes in systolic LV functions did not improve short-term after euglycemia was achieved (after 72 h) and remain unchanged even after longer period (three months) of good glycemic control.

### LV diastolic dysfunction in diabetic patients

Previous investigations have been suggested that in patients with diabetes cardiac dysfunction (such as LV diastolic dysfunction, but also LV systolic dysfunction), defined as separate entity “diabetic cardiomyopathy”, could be detected very early in the clinical course of diabetes, independent of other cardiac risk factors (coronary artery diseases or hypertension) which could progress to heart failure [19, 20]. In that context, echocardiographic studies confirm early diastolic dysfunction in asymptomatic T2D patients [21] which correlate with the level of HbA1c and that a 1% increase in HbA1c was associated with 8% increase of the heart failure risk, independent of other CV risk factors [22]. On the other hands, studies in children and adolescents with T1D found diastolic dysfunction despite intensive insulin treatment and good metabolic control [23–25] demonstrating the existence of high risk for cardiac dysfunction in those patients, independent of the HbA1c level. Similarly, 4 month of strict glucose control in T2D patients, insulin based, does not improve the parameters of diastolic dysfunction in T2D [26]. Moreover, important echocardiographic changes in diastolic function were observed even in patients with prediabetes [27] suggesting that metabolic milieu in T2D, not only chronic hyperglycemia, could influence the cardiac diastolic function. We found in our study that both groups of diabetic patients, with poor and good metabolic control (both groups A and B), have similar changes in E velocity and E/A ratio implying the presence of diastolic dysfunction (regardless of HbA1c level) in comparison to healthy controls. Interestingly, according to our results, acute hyperglycemia does not additionally impair observed LV diastolic dysfunction in these groups of diabetic patients, except the identified changes in transmitral DT which is slightly, but not significantly increase compared to controls. After euglycemia was achieved, DT significantly decline implying that changes in LV diastolic filling patterns might be one of the consequences of acute hyperglycemia in diabetes.

### Layer-specific LV systolic dysfunction in diabetic patients

Strain analysis derived from 2D-STE enables detection of subclinical myocardial systolic dysfunction beyond conventional LV echocardiographic assessment in all three myocardial layers. Studies on layer-specific LV multidirectional strain reported impairments in both longitudinal and circumferential strains in asymptomatic T2D patients which correlated with diabetes duration [28] or overweight [29, 30], while others found longitudinal systolic dysfunction and impairment associated with subendocardial wall thickening, but in poorly controlled asymptomatic and normotensive T2D patients [31]. In addition, STE-derived layer-specific cardiac dysfunctions correlate with the markers of histological remodeling (hypertrophy and fibrosis) and with progression of untreated diabetes in animal models [32, 33]. Using more accurate and sensitive technique, contrast-enhanced cardiovascular magnetic resonance, layer-specific subclinical systolic dysfunction was detected in uncomplicated T2D patients which was associated with impaired coronary microvascular perfusion [34]. Also, it was shown in asymptomatic T2D patients with good glycemic control presence of impairment of LV longitudinal strain but preserved circumferential and radial strain [35], as well as changes in GLS which could be important prognostic factor for the future CV event [36, 37]. In contrast, in 390 patients with chronic ischemic cardiomyopathy (25% with diabetes) endocardial circumferential strain detected by 2D-STE appears to be a better predictor of future cardiac events than total myocardial scar assessed by cardiac magnetic resonance imaging [38]. Very recently, detail analysis of LV mechanics in asymptomatic, hypertensive diabetic patients revealed lower longitudinal strain in Endo layer and lower circumferential strain in Endo and Mid layer compared to healthy controls, which also correlated significantly with HbA1c levels [39]. However, in the study of T2D patients, stratified according to the overall metabolic risk factors, patients with well-control risk factors (including near-normal level of HbA1c), similar to our group B, had only LV diastolic impairment, but there was no evidence of structural or systolic changes of the LV function detected by strain analysis [40].

### Effect of acute hyperglycemia on layer-specific LV strain in diabetic patients

In our group of asymptomatic, normotensive diabetic patients with strict metabolic control (mean HbA1c 6.1 ± 1.0%; Group B) we did not find any statistically significant differences in GLS, peak longitudinal or circumferential strain in comparison to healthy controls (Table 4, Fig. 3). In contrast, in diabetic patients with really poor metabolic control and acute hyperglycemia (Group A, mean HbA1c 11.5 ± 0.3% and glycaemia 22.5 ± 1.1 mmol/l) we found significantly lower GLS, peak longitudinal strain in Endo, Mid and Epi layer, peak circumferential strain in all three layers at basal LV level and in Epi layer in mid-cavity LV level, compared to healthy controls and diabetic patients with good glycemic control (Table 4, Fig. 3). Thus, these results suggest that acute hyperglycemia in asymptomatic diabetic patients have negative impact dominantly on LV systolic function at all three layers (longitudinal and circumferential strain).

To the best of our knowledge, this is the first study investigating the effect of acute hyperglycemia on LV diastolic and systolic function using 2D-STE in diabetic patients without CVD or hypertension. Our results shown that acute hyperglycemia in asymptomatic diabetic patients induces significant LV systolic changes in multilayer myocardial strain, particularly in GLS, peak longitudinal and circumferential strain which remain unchanged after short-term (after 72 h) and long-term (after 3 months) euglycemia was achieved.

Assessment of the effects of acute hyperglycemia on the LV contractile function in diabetic patients has only been previously the subject of a few research papers. In one study, Nielsen R et al. [41] unexpectedly revealed that short-term hyperglycemia (9–12 h) induced by insulin discontinuation, increased LV contractile function (detected as strain rate) in relatively small number (20) of T2D patients with or without heart failure. This result was contrary to previously findings that hyperglycemia is considered harmful in patients with heart failure, particularly when heart failure coexists with diabetes [42]. However, in this mentioned study [41], after insulin discontinuation the level of blood glucose rise till mean 9.9 ± 2.1 mmol/l in diabetic patients without heart failure, while in our group of asymptomatic diabetic patients hyperglycemia was significantly higher and we analyzed not only global strain (like in Nielsen study) [41], but all three LV myocardial layers which could explain different results.

Some of the previously conducted studies have shown that hyperglycemia following a glucose infusion in diabetic patients with normal LVEF, had no effect on the global LV function [43] detected by standard echocardiography, while we used more sophisticated method (strain analysis) for detection of LV systolic function. Studies in animal models indicated that acute hyperglycemia could suppress LV diastolic function and influences mitochondrial energy signaling [44], or induces changes in polyol metabolic pathway with consequently increase in oxidative stress leading to myocardial contractile dysfunction [45]. Compelling evidence is accumulating which suggests a role of oxidative stress as a pathogenic factor underlying the negative effect of acute hyperglycemia on cardiovascular system [9] which could partly explain our results.

Finally, according to our result, the observed changes in LV systolic function during acute hyperglycemia are not reversible neither after short-term (72 h) nor after longer (3 months) period of good glycemic control, although some studies have shown that amelioration in glycemic control over a 1 year period led to improvements in LV systolic function [46]. Several studies suggest the concept that negative early effect of hyperglycemia on cells in diabetes is remembered, the phenomenon named “metabolic memory”, primarily based on mitochondrial damages, overproduction of reactive oxygen species and oxidative stress [47]. Considering that phenomenon, it could be possible that the risk of hyperglycemia-related vascular complications in diabetes persist even when hyperglycemia is normalized, as previously shown in long-term (several years) trials [48, 49]. However, in our study, the follow-up period with good glycemic control was only three months which possibly would not be long enough to see improvement in the observed LV systolic dysfunction.

## Research limitations

The present study has several possible limitations. First, our study population is relatively small and consists of both T1D and T2D patients, but the separate analysis has shown the same results, regardless of diabetes type. Thus, results are presented for whole diabetes patients group and control diabetes group (Group B) was adjusted for type of diabetes. Second, we could not exclude subclinical coronary artery disease in our diabetes patients because stress test was not performed. Third, due to the GE EchoPAC version 113 software limitations, the images are two-dimensional, and once the ‘dots’ on the image ‘move out’ of the plane during a cardiac cycle they cannot be monitored by this software.

## Conclusion

The acute hyperglycemia in asymptomatic diabetic patients with preserved LVEF have significant negative effects on systolic LV myocardial mechanics primarily by reducing GLS, peak systolic longitudinal (at Endo, Mid and Epi layers) and peak circumferential strain (at all three layers in basal LV level). Moreover, observed changes in systolic LV functions was not reversible after three months of good glycemic control.

## Data Availability

The datasets generated and analyzed for this study are available from the corresponding author upon reasonable request.

## Abbreviations

2D-STE: two-dimensional speckle tracking echocardiography
AMI: acute myocardial infarction
BMI: body mass index
Ch: cholesterol
CVD: cardiovascular disease
DT: deceleration time
EDV: end-diastolic volume
EF: ejection fraction
Endo: subendocardial layer
Epi: subepicardial layer
ESV: end-systolic volumes
GLS: global longitudinal strain
ICC: interclass correlation coefficient
ICU: Intensive Care Unit
LAVI: left atrium volume index
LV: left ventricle
Mid: mid-myocardial layer
NT-proBNP: N terminal brain fragment (B-type) for natriuretic peptide
T1D: type 1 diabetes
T2D: type 2 diabetes
